# Factors associated with academic resilience in disadvantaged students: An analysis based on the PISA 2015 B-S-J-G (China) sample

**DOI:** 10.3389/fpsyg.2022.846466

**Published:** 2022-09-29

**Authors:** Songli Jin, Guangbao Fang, Kwok Cheung Cheung, Pou Seong Sit

**Affiliations:** ^1^Institute of Educational Innovation, Chongqing University of Arts and Sciences, Chongqing, China; ^2^School of Education, Fujian Normal University, Fuzhou, China; ^3^Faculty of Education, University of Macau, Macau, Macau SAR, China

**Keywords:** academic resilience, classification and regression tree, disadvantaged students, program for International Student Assessment, scientific literacy

## Abstract

Academic resilience is evident in students who are living in vulnerable environments, yet achieve success in academic outcomes. As a result, substantial attention has been devoted to identifying the factors associated with academic resilience and supporting students to be resilient. This study used the Classification and Regression Tree and Multilevel Logistic Regression modeling to identify the potential factors related to students’ academic resilience. Using these tools, the study analyzed the B-S-J-G (China) sample in PISA 2015. The variables that significantly predicted whether a student is disadvantaged and resilient (DRS) or not resilient (DNRS) were shown to be: *Proportion of teachers in school with master’s degrees, Proportion of teachers in school with bachelor’s degrees, Environmental awareness, Science learning time per week, Number of learning domains with additional instruction, and Students’ expected occupational status*. These findings may enlighten governments, teachers, and parents on ways to assist students to be resilient.

## Introduction

Socioeconomic status (SES) is highly associated with students’ academic achievement (White, 1982; Sirin, 2005; Wang, 2009; Ren and Xin, 2013), suggesting that students with higher SES are more likely to outperform their classmates. However, some students from low-SES households attain high levels of academic success. These children are designated as Disadvantaged Resilient Students (DRS) because they are able to overcome the negative effects of their adverse circumstances and achieve educational success beyond the predicted SES-based outcomes (Cheung, 2017). In addition, there is a subset of pupils known as Disadvantaged Non-resilient Students (DNRS) who are from households with low socioeconomic status and have low academic achievement. Moreover, as digital natives (Prensky, 2001) who grew up with technology, the millennial generation has been compelled to increase its scientific literacy in order to adapt to the current society. In recent decades, this demand for enhancing pupils’ scientific literacy has received considerable attention (Chang, 2015). How teachers, parents, and educational policymakers can assist children from low socioeconomic backgrounds to overcome their adverse situations and develop resilience in science learning is a crucial challenge for educators. The first step in answering this question is to identify the potential factors that are strongly related to students’ resilience in scientific literacy performance.

PISA 2015 provides an opportunity to address this problem in the domain of scientific literacy. PISA was developed by the Organization for Economic Co-operation and Development (OECD) to assess 15-year-old students’ literacy in the fields of science, mathematics and reading needed for full participation in modern societies. Assessments occur every 3 years in many regions of the world. 72 countries and economies participated in the 2015 PISA. The main domain of PISA 2015 was science, thereby providing a comprehensive measure of student performance in this domain.

## Literature review

### Conceptual framework

Walberg (1981) proposed the education productivity theory, which asserts that students’ learning is inextricably linked to their social settings. The social context was further defined in a series of studies (Walberg, 1984), as nine elements classified into three groups. The first is about student aptitude, which encompasses ability, development, and motivation. The second category is concerned with instruction and is comprised of two components: instructional quality and quantity. Finally, there is the category of environment, which includes the home, classroom, peer group, and mass media-environments (Walberg, 1986; Fraser et al., 1987). Furthermore, the education productivity theory considered that the influences of all these elements on students’ learning should be studied holistically, rather than individually, because their effects are more apparent when combined (Chen et al., 2021). Therefore, consistent with the education productivity theory, this study investigated factors associated with students, family, and schools overall, as well as prospective factors associated with students’ academic resilience and scientific literacy performance.

**FIGURE 1:**
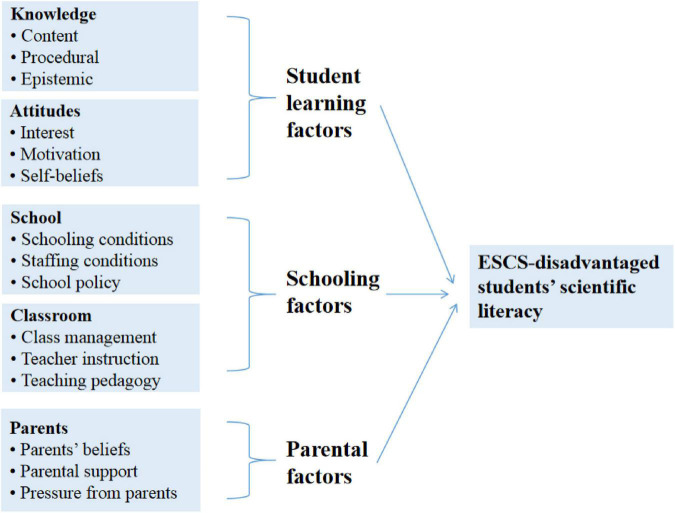
Conceptual framework of factors associated with the scientific literacy performance of economic, social and cultural status (ESCS)-disadvantaged students.

Scientific literacy was defined in the PISA 2015 as the capacity to engage as a reflective citizen in issues and concepts related to science, and included three specific competencies involving being able to: scientifically explain phenomena, evaluate and design scientific enquiry, and scientifically interpret data and evidence (Organization for Economic Cooperation and Development [OECD], 2016a). PISA 2015 provides a framework for identifying probable factors affecting pupils’ scientific literacy. According to the PISA 2015 framework for assessing scientific literacy, students’ scientific literacy is related to three types of knowledge (content, procedural, and epistemic knowledge), students’ attitudes toward science (such as students’ interest in science and environmental awareness), and context variables (personal, local/national, and global contexts) (Organization for Economic Cooperation and Development [OECD], 2016a). Accordingly, PISA 2015 took a holistic and comprehensive approach to explaining how students’ scientific literacy developed. Based on scrutiny of the educational productivity theory and the PISA 2015 framework on scientific literacy, this study concluded that these two frameworks emphasized the importance of personal and local elements, which are students-related, parent-related, teacher-related, and school-related factors in the educational context. Given the overlap of explanatory factors associated with students’ learning (in their scientific literacy performance), this study created a new framework (see Figure 1) by combining the education productivity theory and the PISA 2015 scientific literacy assessment framework to guide the research design and selection of variables that could affect the performance of PISA index of economic, social and cultural status (ESCS) disadvantaged students in science literacy.

### Factors associated with students’ academic resilience in scientific literacy

Academic resilience is regarded as a characteristic of the students with low social-economic status who have achieved outstanding academic outcomes (Organization for Economic Cooperation and Development [OECD], 2011). Although PISA alternates its main domain between reading, mathematics, and science every 3 years, the discriminating factors affecting students’ academic resilience may be similar across these three domains, as some students who exhibit resilience in one domain are likely to demonstrate resilience in others (Organization for Economic Cooperation and Development [OECD], 2011). Moreover, the majority of existing research ignores the specific domains of students’ resilience when examining the elements that contribute to students’ academic resilience. These two findings may indicate that the research in other domains may have relevance for our work. Therefore, this study included research in the domains of mathematics and reading to indicate the possible factors associated with students’ academic resilience.

On the students’ level, their enjoyment of learning a subject, metacognitive awareness of learning strategies, and participation in a variety of learning activities all correlated positively with students’ academic resilience, such as in reading (Shen, 2012; Cheung et al., 2014), and in mathematics (Alivernini et al., 2016; Cheung, 2017). Additionally, Clavel et al. (2021) found a strong association between students’ enjoyment and interest in science and their academic resilience in science. Furthermore, the analysis of PISA 2015 revealed that students’ epistemic beliefs about science, learning time, and science self-efficacy are all positively associated with students’ resilience in science (Alivernini and Manganelli, 2015; She et al., 2019). Agasisti et al. (2016) indicated that students in a class whose peers have higher academic achievements are more likely to be resilient. Similarly, Cordero and Mateos-Romero (2021) suggested that students’ learning skills prior to entering school, as well as their primary school classmates’ socioeconomic status, are strongly associated with their academic resilience, based on an analysis of TIMSS (2015) and PIRLS (2016) data. In addition, Agasisti et al. (2021) indicated that students who attend schools with a supportive disciplinary climate, and receive additional time for instruction in critical areas are more likely to develop resilient capabilities. Agasisti and Longobardi (2017) argued in another study that if schools could provide more extracurricular activities for students, they would be more resilient. A comparable study discovered that for the African American women they studied, experiences outside of school were more critical than experiences within their schools for building up resilience (Ferguson and Martin-Dunlop, 2021). Chirkina et al. (2020) found that students’ attitudes toward mathematics, their general test scores, and the average school social economic status and school type, are significantly correlated with their academic resilience. Alivernini and Manganelli (2015) asserted that teachers’ salaries, parental pressure on schools, and school size are all associated with students’ resilience in science.

### Factors associated with students’ science performance

This study categorizes the factors associated with students’ scientific literacy performance into three areas, namely student-related, school-related, and family-related components. On the student level, the enjoyment of science learning was identified as the strongest factor in students’ scientific performance (Altun and Kalkan, 2021; Lau and Ho, 2022). Kalkan et al. (2020) revealed that male students’ scientific performance was much higher than female students in Turkey, Singapore, the United States, Italy, and Brazil. This finding is consistent with the results of various other studies, which showed that male students outperform female students (Sun et al., 2012; Lam and Lau, 2014; Chi et al., 2018). Moreover, Lau and Ho (2022) argued that, compared with male students, female students have lower science performance and less positive attitudes toward science with an international sample. In addition, studies have also reported that students’ lack of motivation when learning science increases their possibility of low achievement in science (Glynn et al., 2007; Areepattamannil et al., 2011). Chen et al. (2021) also stated that students’ science self-efficacy is a positive significant factor in students’ science performance. This finding is also echoed in the research of Alatli (2020).

On the teacher level, it has been demonstrated that teacher shortage is negatively connected with students’ scientific literacy performance in many countries, such as in Brazil (Kalkan et al., 2020), in Turkey and Singapore (Alatli, 2020), and in Finland (Nissinen et al., 2018). By studying PISA 2015 data, Ilgaz et al. (2019) extended this negative relation to 70 countries. Furthermore, teachers’ teaching methods have a strong correlation with students’ science performance. For example, Lau and Ho (2022) revealed that teachers’ teaching practices, direct teaching, and adapted instruction are positively associated with students’ enjoyment of scientific learning and performance (Alatli, 2020; Chen et al., 2021). In addition, it has been contended that teachers’ experiences and engagement in professional development activities are positively associated with students’ science performance (Wenglinsky, 2002; Blank and De Las Alas, 2009). By contrast, You et al. (2020) argued that teachers’ teaching experience, and engagement in professional development have no discernible relationships with students’ scientific literacy.

Regarding the school level, You et al. (2020) stated that school-level factors might account for 21% of the variance on students’ scientific literacy. Previous research revealed a variety of factors related to students’ scientific literacy performance, including the school disciplinary climate (Altun and Kalkan, 2021), school leadership, and instructional resources (Areepattamannil et al., 2015; Topcu et al., 2015; Chi et al., 2018; Chen and Cui, 2019; Chen et al., 2021).

The relationship between school resources and students’ achievements in science is still inconclusive. On one hand, some studies have indicated that school resources, such as those devoted to enhancing classroom conditions or teacher quality, show no substantial association with students’ science performance (Hanushek, 1996), or any direct positive effect on their achievements (Hanushek, 1997; Picus et al., 2005). On the other hand, a review of the research insisted that school resources positively related to students’ achievements in various subjects (Greenwald et al., 1996). In terms of the school’s mean socioeconomic status, research has indicated that this correlates positively with students’ achievements, including their science performance (Perry and McConney, 2010; You and Delgado, 2015).

On the basis of the literature reviewed above, this study concludes that few studies have incorporated all of these viewpoints; nor have they found which variables have a greater impact on students’ science literacy.

## Research questions

Therefore, this study used the sample of B-S-J-G (China) to address these gaps, the research questions is: What are the potential variables underlying the distinctions between the DRS and DNRS in the sample of B-S-J-G (China) in PISA 2015?

## Materials and methods

### Sample

Using the B-S-J-G (China) data from the PISA 2015 database, which is publicly accessible *via* the official website of the OECD, this study seeks to address the research objective outlined above. There were 9841 students from B-S-J-G (China) in the initial sample, including 4682 females and 5159 males. This study’s sample consisted of 2,450 DRS and DNRS aged 15 from B-S-J-G (China), with 1168 female and 1282 male disadvantaged pupils. These students are positioned in the lowest quarter of the socioeconomic status distribution, as defined by the PISA index of ESCS; they are referred to as home-disadvantaged pupils in the current study. Technically, the DRS and DNRS are identified through three phases that are consistent with the method used in the PISA report to identify resilient students (Organization for Economic Cooperation and Development [OECD], 2016b).

The first stage is to identify disadvantaged students whose ESCS falls inside the bottom quarter of the B-S-J-G (China) ESCS distribution. Among the 9841 students of B-S-J-G (China), 2450 have been identified as disadvantaged students.

Next, the observed student scores on scientific literacy were regressed on the student ESCS across all participating countries/economies to establish the international performance-ESCS regression line. This regression line calculates anticipated student scores on the scientific literacy test. It is noteworthy that this study selected the first plausible value from the ten plausible values of science literacy in the PISA database as the observed scientific literacy score, because using one plausible value or all plausible values does not make a significant difference on large sample sizes (Organization for Economic Cooperation and Development [OECD], 2009).

**FIGURE 2:**
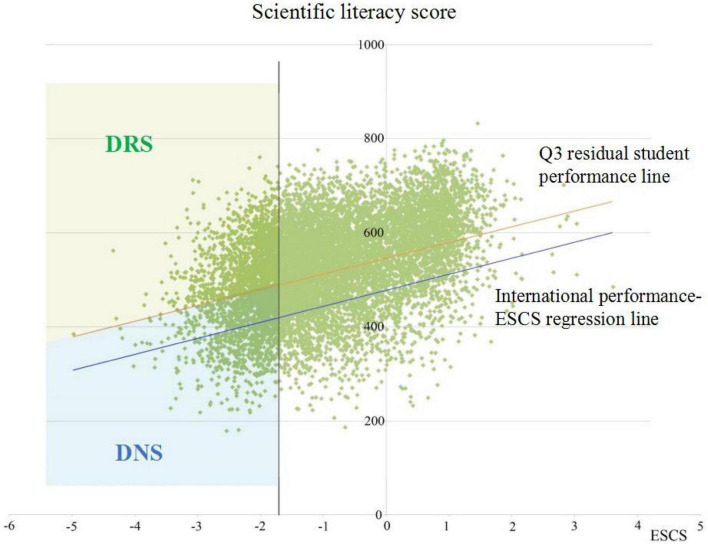
Identification of disadvantaged and resilient (DRS) and disadvantaged non-resilient students (DNRS) in the PISA 2015 B-S-J-G (China) sample.

Finally, the residual scores of pupils are calculated by subtracting their observed scientific literacy scores from their expected scientific literacy ratings. If students’ residual scores exceed the international top quarter residual, they will be classed as DRS, and if their residual scores fall below the international top quarter residual, they will be categorized as DNRS (see Figure 2). According to this classification, there are 1186 (48.4%) DRS and 1264 (51.6%) DNRS in the B-S-J-G (China) sample for further research in this study. In particular, the DRS should meet the two following criteria: (1) Their ESCS is in the lowest quartile of B-S-J-G (China) ESCS’s distribution, and (2) Their residual performance exceeds the international top quartile residual performance.

### Variables

The dependent variable is the classification of resilient and non-resilient ESCS-disadvantaged students. According to the PISA, academically resilient children are those who come from families with a low socioeconomic position but yet obtain better results than expected (Organization for Economic Cooperation and Development [OECD], 2016b).

PISA collects a multitude of variables. In accordance with the conceptual model (Figure 1). Appendix 1 presents the independent variables evaluated in this study. These variables are obtained from the PISA 2015 tests as well as student, school, and parent questionnaires.

The modular structure of the PISA 2015 questionnaires contains two rows of topics separated into two portions: science-related topics and general topics. Science-related topics consist of the learning environments at the school level that explicitly promote science education, such as laboratories, science-related education curriculum, collaboration among science professionals, and the values ascribed to science by the school community. The modular structure of PISA 2015 summarizes student background characteristics and science learning processes, respectively. In particular, the student background variables are associated with family and family members’ education, whereas the processes are associated with three themes for in-depth examination (i.e., teaching and learning, school policies, and governance). The modular structure of PISA 2015 also discusses the non-cognitive outcomes of education (e.g., motivation, interest, beliefs, and career aspirations). The present study selected factors in accordance with the conceptual model (Figure 1) and the modular structure of the PISA 2015 questionnaires.

### Data analysis methodologies

This study utilized the Classification and Regression Trees (CART) and Multilevel Logistic Regression (MLR) to analyze data. CART provides various benefits over other classification and regression techniques. First, it can analyze tens of thousands of nominal, ordinal, and continuous independent variables with varying degrees of measurement. In addition, no assumptions are made about the distribution of the independent variables. Second, multicollinearity between independent variables has no effect on CART. It is a data mining method that evaluates a vast variety of predictor variables and is unaffected by the multitude of complex interactions between them. Therefore, some studies involving a large number of predictor variables have applied CART for data analysis of international educational data sets. For example, Alivernini (2013) used CART to identify variables (at country, school and student levels) associated with the differences of highest and lowest ability readers based on the Progress in International Reading Literacy Study (PIRLS) 2006 data. Sanzana et al. (2015) study identified groups of eighth-grade elementary students according to their performance in the mathematics test, using features related to individual and family behavior through random forest (RF) and CART with a database provided by the Education Quality Measurement System of Chile. Moreover, Liu and Ruiz (2008) predicted K-12 students’ competence levels on test items related to energy using data mining algorithms similar to CART, based on data sets of the Third International Mathematics and Science Study (TIMSS; 1995, 1999, and 2003) and the National Assessment of Educational Progress (NAEP). Third, the advanced algorithms used in CART can effectively manage missing data. Fourth, the results are reasonably straightforward to interpret (Breiman et al., 1998; Allore et al., 2005; Strobl et al., 2009).

However, the main problem with CART is that the sample in a subset of the analysis are the students in this subset instead of the whole sample. If the study needs to check the factors associated with the differences between the DRS and the DNRS in relation to the whole sample, MLR would be an appropriate choice. MLR is well-suited for describing and testing hypotheses about relationships between a categorical outcome variable and several predictor variables with the whole sample (Peng et al., 2002). Therefore, this study employs MLR to expand upon the findings of CART analysis. In the present study, WesVar 5.1 software was employed to conduct MLR using replicated weights and complex design weights for an unbiased estimation of the parameters (WESTAT, 2007).

This study employed the Gini index as the statistical criterion for terminating successive CART iterations and was conducted with SPSS 26.0. Based on the idea of producing the most homogeneous groupings, CART automatically selects the most influential partitioning variable from the independent variables. The target sample (the parent node) is separated into two homogenous subgroups (the child nodes) depending on a particular independent variable. Afterward, each of the child nodes are separated into two subgroups using the same technique. This procedure is repeated until the impurity reduction satisfies a predefined criterion (Gini index.001) or the number of students in a subgroup falls below a predetermined threshold, which in this study was set at 50 (Strobl et al., 2009). In this study, a maximally homogenous node includes students who are either DRS or DNRS. Cross-validation procedures are then utilized to confirm the results.

## Results

### Factors related with the classification of academically resilient students

**FIGURE 3:**
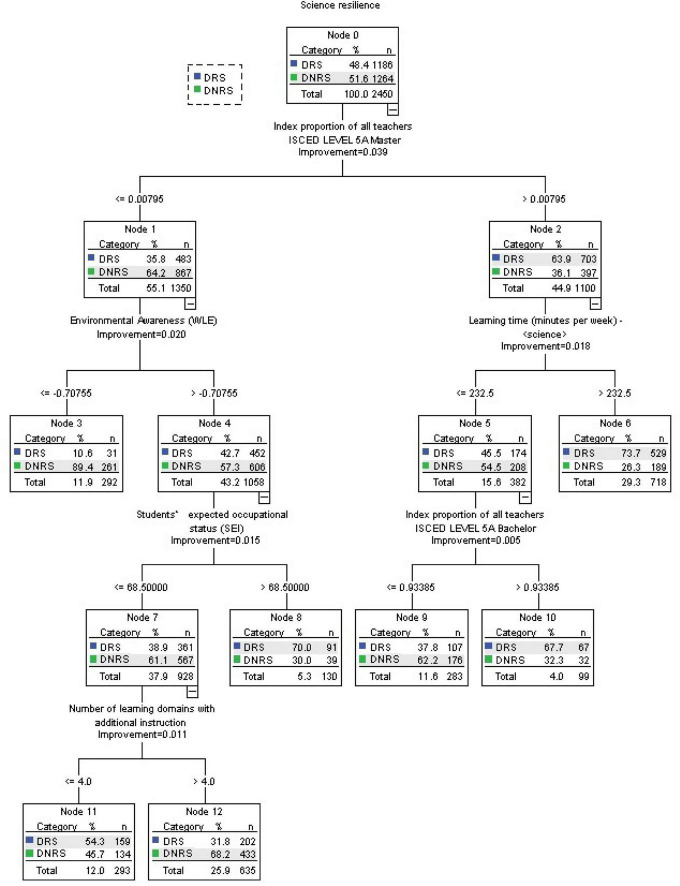
Results of the Classification and Regression Trees (CART) analysis for the B-S-J-G (China) data.

Figure 3 demonstrates the classification tree generated using CART. It consists of seven terminal nodes (subgroups): node 3, node 6, node 8, node 9, node 10, node 11, and node 12. Students inside nodes 6, 8, 10, and 11 are expected to be DRS, whereas students within nodes 3, node 9, and 12 are predicted to be DNRS. The accuracy of the model is estimated using 10-fold cross-validation, which is superior to other cross-validation techniques with fewer iterations (Breiman et al., 1998).

The most important variable among the 81 variables is the *Proportion of teachers in school with master’s degrees*. This split improves the Gini index by 0.039. This means the impurity of the target sample (Node 0) is reduced by 0.039. That is to say, the subgroups (Node 1 and Node 2) become more homogeneous. If a school has more than.8% of teachers with a master’s degree qualification (at the 55th percentile of the classifying variable), the probability of ESCS-disadvantaged students at the school becoming resilient rises from 48.4% to 63.9% (node 2). However, if a school has equal to or less than 0.8% of teachers with a master’s degree qualification, there is a possibility that the level of DRS students will go down from 48.4% to 35.8% (node 1).

The classification tree is interpreted from the right to the left. The next variable is *Science learning time per week*. This split improves the Gini index further by 0.018 based on 0.039. It means the impurity of the parent node is reduced by 0.057, and the subgroups (Node 5 and Node 6) become more homogeneous. For students who study at a school with more than 0.8% of teachers holding a master’s degree (on the right-hand side of the tree), and if they learn science for more than 232.5 min per week (at the 35th percentile), the percentage of DRS students rises from 63.9% to 73.7% (Node 6). Also, if students learn science for equal to or less than 232.5 min per week, the percentage of DRS students goes down from 63.9% to 45.5% (node 5).

Furthermore, when students learn science for equal to or less than 232.5 min per week, the variable that determines whether they are DRS or DNRS relates to the *Proportion of teachers in school with bachelor’s degrees*. This split improves the Gini index further by 0.005 based on 0.057, that is 0.062, and the subgroups (Node 9 and Node 10) become more homogeneous. If a school has more than 93.4% of teachers with a bachelor’s degree (at the 77th percentile), the percentage of DRS students in this school rises from 45.5% to 67.7% (Node 10). However, if this school has equal to or less than 93.4%, the percentage of DRS students in this school goes down slightly from 45.5% to 37.8% (node 9).

On the right branch of the tree relating to students who study in a school with equal to or less than.8% of teachers with a master’s degree (in Node 1). This split improves the Gini index further by 0.020 based on 0.039. This means the impurity of the target sample (Node 0) is reduced by 0.059, and the subgroups (Node 3 and Node 4) become more homogeneous. If a student in Node 1 has a high *Environmental awareness* above −0.708 (at the 20th percentile), the probability of him/her being a DRS increases from 35.8% to 42.7% (Node 4); but if this is equal to or lower than −0.708, the probability decreases remarkably from 35.8% to 10.6% (Node 3). Students in Nodes 3 and 4 will be predicted to be DNRS.

However, the percentage of DRS in Node 4 can be improved by the variable of *Students’ expected occupational status*. This split improves the Gini index further by 0.015 based on 0.059, that is 0.074, and the subgroups (Node 7 and Node 8) become more homogeneous. If a student in Node 4 has a high expected occupational status above 68.5 (at the 80th percentile), the probability of him/her rising to a DRS increases dramatically from 42.7% to 70.0% (Node 8); but if it is equal to or lower than 68.5, the probability of him/her becoming a DRS decreases a little from 42.7% to 38.9% (Node 7).

Finally, the variable relating to the *Number of learning domains with additional instruction* affects whether a student is considered as DRS or DNRS in Node 7. This split improves the Gini index further by 0.011 based on 0.074. If the student in Node 7 learns equal to or less than four learning domains of additional instruction (at the 40th percentile), the probability of him/her becoming a DRS increases from 38.9% to 54.3%. However, if the student receives more than four learning domains of additional instruction, the probability of him/her becoming a DRS decreases slightly to 31.8%.

### Factors associated with the differences of disadvantaged and resilient and disadvantaged non-resilient students

This study employed MLR to duplicate and expand upon the findings of CART analysis (Alivernini and Manganelli, 2015). The samples in the child nodes of CART analysis consist only of the students in its parent nodes, not the entire sample (for example, the sample of the analysis to divide Node1 into Node 3 and Node 4 is just 1350 students in Node 1, rather than the entire sample of 2450), so it is necessary to use MLR to test the factor associated with the differences between the DRS and the DNRS in the entire sample.

The dependent variable in MLR is dichotomous (DRS vs. DNRS), and the independent variables are those differentiating variables found in CART, namely, *Proportion of teachers in school with master’s degrees, Proportion of teachers in school with bachelor’s degrees, Environmental awareness, Science learning time per week, Students’ expected occupational status*, and *Number of learning domains with additional instruction*. In the MLR analysis, the average of the school ESCS serves as a control variable. Both the student and school levels employ weights. This study employed the final student weight variable (W_FSTUWT) from the PISA raw data to weight the student level variables and the total of W_FSTUWT within each school as between-school weights for school level analysis in MLR (Organization for Economic Cooperation and Development [OECD], 2016b). The outcome is shown in Table 1.

**TABLE 1:** Results of Multilevel Logistic Regression (MLR) of the disadvantaged and resilient (DRS) vs. disadvantaged non-resilient students (DNRS) classification.

	Variable	*B*	S.E.	Exp (*B*)
	Intercept	1.104	1.594	
School level	Proportion of teachers in school with master’s degrees	9.154*	3.818	9454.813
	Proportion of teachers in school with bachelor’s degrees	1.381**	0.427	3.978
	School ESCS (control variable)	1.596*	0.644	4.934
Student level	Environmental awareness	0.451**	0.102	1.569
	Science learning time per week	0.002**	0.000	1.002
	Students’ expected occupational status	0.028**	0.005	1.029
	Number of learning domains with additional instruction	−0.182**	0.022	0.834

(1) ** <0.01, * <0.05. (2) Negative log-likelihood = 0.209; Likelihood ratio (Cox-Snell) = 0.251; Likelihood ratio (Estrella) = 0.277. (3) Variable is at school level or at student level is according to PISA 2015 technical report (Organization for Economic Cooperation and Development [OECD], 2017).

The results indicate that the six essential factors show adequate effect in predicting whether a student is a DRS or a DNRS, with Negative log-likelihood, Cox-Snell, and Estrella indices ranging from 0.209 to 0.277 (WESTAT, 2007). According to Table 1, teacher qualifications are of utmost importance in B-S-J-G (China), since an increase of one standard deviation in the *Proportion of teachers in school with master’s degrees* results in a 9455-fold increase in the likelihood that a pupil is academically resilient. There is a 4-fold rise for teachers with bachelor’s degrees. An increase of one standard deviation in *Environmental awareness* improves the likelihood that an ESCS-disadvantaged youngster will be academically resilient by 56.9 percent. An increase of one standard deviation in the *Number of learning domains with additional instruction* reduces the likelihood that an ESCS-disadvantaged student will be academically resilient by 16.6 percent. For a one-unit increase in the standard deviation of the indices, the influence of the *Science learning time per week* and the *Students’ expected occupational status* is 0.2% and 2.9%, respectively.

To summarize, the six variables found to significantly predict whether a student is a DRS or a DNRS are: *Proportion of teachers in school with master’s degrees, Proportion of teachers in school with bachelor’s degrees, Environmental awareness, Science learning time per week, Number of learning domains with additional instruction, and Students’ expected occupational status*. *Environmental awareness, Science learning time per week, and Students’ expected occupational status* are literacy learning factors. *Proportion of teachers in school with master’s degrees* and *Proportion of teachers in school with bachelor’s degrees* are schooling factors; and, *Number of learning domains with additional instruction* is considered in this present study as both learning and parental factors.

## Discussion and conclusion

### Discussion

This study has sought to elucidate the various characteristics in their family, personal and educational backgrounds that differentiate DRS from DNRS adolescents in scientific literacy. This study suggests that the number of teachers with master’s or bachelor’s degrees in schools could significantly increase the probability of academic resilience on scientific literacy of ESCS-disadvantaged children, a finding that is supported by numerous other studies (Goldhaber and Brewer, 1997, 2000; Clotfelter et al., 2007; Chu et al., 2015). In a research review, Wayne and Youngs (2003) offered a possible reason for this link, as teachers with advanced degrees are more likely to teach more effectively and offer their students more learning supports.

This study also indicates that students’ environmental awareness could add their possibility of been resilience in scientific literacy. This study’s findings are congruent with other studies (Bybee, 2008; Organization for Economic Cooperation and Development [OECD], 2016a). In the PISA 2015, awareness of environmental issues was one important aspect of the construct attitudes toward science (Organization for Economic Cooperation and Development [OECD], 2016a). According to Bybee (2008), the scientific literacy of students should be developed in parallel with their attitudes and beliefs regarding natural resources and the quality of the environment. Natural resources and environmental quality are two major areas in which scientific literacy has significant importance for promoting and preserving the quality of life and formulating public policy for individuals and communities (Organization for Economic Cooperation and Development [OECD], 2016a). Therefore, environmental awareness is an essential component of ESCS-disadvantaged students’ scientific literacy.

This study presents that spending more than 4 h per week studying science is significantly related with the ESCS-disadvantaged students’ resilience in scientific literacy, while the regression coefficient is modest. Agasisti et al. (2021) have also demonstrated that students are more likely to develop resilience when students were given additional time for instruction in essential courses. Because of the exam-based feature of high school courses in China, many Chinese high school students devote the majority of their time to study (Leung, 2021). This study reveals that 65 percent of B-S-J-G (China) ESCS underprivileged students who study science for more than 4 h per week are likely to be marginally more academically resilient in scientific literacy.

To our knowledge, there is no direct evidence to confirm the positive relationship between students’ career expectations and their resilience in scientific literacy. This research filles this gap by indicating that the possibility of students be resilient in science literacy would increase when students have a higher expected occupational status. Moreover, as some research suggested, students’ career expectation is positively related with their learning motivations (Domene et al., 2011), and students’ science learning motivation is an essential component affecting their science accomplishment (Glynn et al., 2007; Areepattamannil et al., 2011). Therefore, students’ learning motivation may mediate the relationship between students’ career expectation and their academic resilience in scientific literacy, which requires to be assessed in the future study.

The number of learning domains in which ESCS-disadvantaged pupils receive additional instruction has a negative relationship with their probability of be resilient in scientific literacy. The potential reason of this relationship may be that students spent so much time on learning other subjects, such as mathematics, and English, which are critical in College Entrance Examination (高考) (Zhang, 2011; Zhang and Bray, 2018) rather than in science. For example, Zhang and Bray (2018) indicated that 58.7 percent of sampled students in Grades 3–9 in Shanghai had received various additional instruction, while 81.5 percent and 76.8 percent of those students received tutoring in Mathematics and English, respectively. Similarly, this study finds that 60 percent of ESCS-disadvantaged students in the B-S-J-G (China) sample participated in more than four learning domains requiring additional instruction. There is a possibility for these students that they may not have sufficient time to learning science.

In addition, receiving excessive amount of additional instruction may increase students’ academic pressure, resulting in a decline in instructional efficacy (Št’astný et al., 2021). As indicated by Huang and Chen (2008), the link between the quantity of additional instruction and academic performance is not a positive and linear relationship; rather, it follows a “climb first and then fall” pattern. To some extent, additional instruction may improve students’ academic performance, whereas excessive additional instruction definitely have negative influences on students’ learning. Thus, when ESCS-disadvantaged pupils participated in excessive additional instruction, their scientific literacy performance would decline and be non-resilient.

### Implications

This exploratory study aimed to identify the influential predictors of whether a student is a DRS or DNRS in four prosperous Chinese cities/provinces. The PISA 2015 data offer a wealth of information and can be investigated in depth through educational data mining to reveal important knowledge to bolster informed policy-making. This research illustrates that it is possible to obtain information with crucial significance. Important characteristics that predict the classification of pupils as DRS or DNRS in the other PISA 2015 participating economies may or may not be identical to those discovered for B-S-J-G (China). However, the most significant features discovered may indicate to researchers and educational practitioners in various educational systems the way forward in terms of enhancing the scientific literacy of ESCS-disadvantaged pupils.

This study has suggested that the proportion of teachers with master’s or bachelor’s degrees in schools is related to the students’ resilience in scientific literacy. In order to effectively improve the students’ resilience in scientific literacy, it is necessary to increase the academic qualifications of teachers. Understanding this is crucial for the B-S-J-G (China) Governments and school administrators, since on the basis of this knowledge they can provide instructors with more opportunity to advance their education and achieve higher academic levels.

In addition, this study indicated that environmental awareness is a significant element related to the DRS’s scientific literacy. Considering the significance of environmental challenges to the sustainability of life on Earth and the survival of humanity, the OECD has suggested that young people must learn to plan their lives in accordance with ecological principles (Organization for Economic Cooperation and Development [OECD], 2016a). Thus, fostering awareness of environmental issues and a responsible attitude toward the sustainability of the environment are essential for modern science education.

In recent years, a growing number of Chinese policymakers and academics have focused on natural resources and environmental quality (e.g., Stalley and Yang, 2006; He et al., 2011; Lumkes et al., 2012; Wu, 2013). However, schools in different districts place varying amounts of emphasis on environmental education and implement it in various ways. Consequently, children in B-S-J-G (China) acquire various types of environmental education and achieve varying academic levels in this subject at their respective schools. The B-S-J-G (China) Governments must review the implementation of environmental education in schools so that each student receives a high-quality environmental education. Students must also share responsibilities for fostering environmental awareness.

This study found that spending more than 4 h per week studying science is connected with students’ resilience in scientific literacy. However, time is only one aspect of the equation; learning efficacy is the other. Throughout their studies, ESCS-disadvantaged students must guarantee that their learning is effective. Educatively relevant teacher scaffolding is required in this regard.

If a student’s expected occupational status is above the 80th percentile of his or her peers in B-S-J-G (China), he or she will have a greater chance of being categorized as a DRS rather than a DNRS. The predicted occupational position of Chinese pupils may be affected by their self-evaluation, parental background, expectations, as well as social appraisal and support from instructors, peers, parents, and relatives (Hsieh, 2005; Wang, 2007). Therefore, instructors, parents, classmates, and relatives are encouraged to provide children and adolescents with accurate evaluations and to encourage them to reach a higher occupational standing.

This study indicates that excessive additional education may negatively impact students’ resilience in scientific literacy performance. Due to cost constraints, parents in China determine the extent to which ESCS-disadvantaged students receive additional teaching in domain courses learned at school. According to the findings of the present study conducted in B-S-J-G (China), parents should not force their children to take on more than four learning domains at any one time. China has introduced a “double reduction” strategy in 2021 to alleviate the strain of excessive homework and off-campus tutoring for compulsory education students (Xu and Jianli, 2021).

### Limitation

In this study, CART and MLR were used to identify the significant factors of the DRS to DNRS classification of teenagers in B-S-J-G (China) based on the assessment of scientific literacy. However, this cannot depict the intricate interactions between these significant components. Consequently, future research could investigate the links between these variables using structural equation modeling or other causal modeling techniques.

### Conclusion

Based on the education productivity theory, this study utilized the B-S-J-G (China) data from the PISA 2015 database to investigate the relationship between student-related, school-related, and parental factors by differentiating between DRS and DNRS pupils. The CART and MLR were used to determine the distinguishing factors. According to the findings of this study, the following variables significantly predicted whether a student is a DRS or DNRS are: *Proportion of teachers in school with master’s degrees, Proportion of teachers in school with bachelor’s degrees, Environmental awareness, Science learning time per week, Number of learning domains with additional instruction*, and *Students’ expected occupational status*. These findings also enlighten governments, educational practitioners, and parents about ways to assist DNRS youth in attaining a greater level of scientific literacy.

## Data availability statement

Publicly available datasets were analyzed in this study. This data can be found here: OECD-PISA2015 China dataset, https://www.oecd.org/pisa/data/2015database/.

## Ethics statement

Ethical review and approval was not required for the study on human participants in accordance with the local legislation and institutional requirements. Written informed consent from the participants’ legal guardian/next of kin was not required to participate in this study in accordance with the national legislation and the institutional requirements.

## Author contributions

SJ: writes the raw manuscript. GF: revises and proofreads the manuscript. KC and PS: supervise the writing of the raw manuscript. All authors contributed to the article and approved the submitted version.

## Appendix

**TABLE A1:** Independent variables drawn from the PISA 2015 database.

Variable type	Classification	Variable label
Personal factors	Student background	Gender
		Grade repetition
		Duration in early childhood education and care
		Number of school changes
		Number of changes in educational biography
Learning factors	Knowledge and experience	Index of science activities
		Perceived feedback
		Adaption of instruction
		Number of learning domains with additional instruction
		Total hours of additional instruction
		Number of science disciplines and subjects with additional instruction
		Out-of-school study time per week
		Science learning time per week
		Learning time per week in total
		Child’s past science activities
		Student behavior hindering learning
		ICT use outside of school for schoolwork
		ICT use outside of school leisure
		Students’ perceived ICT competence
		Students’ ICT as a topic in social interaction
		Students’ perceived autonomy related to ICT use
	Attitudes and beliefs	Student attitudes, preferences and self-related beliefs: Achieving motivation
		Collaboration and teamwork dispositions: Enjoy cooperation
		Collaboration and teamwork dispositions: Value cooperation
		Students’ ICT interest
		Students’ expected occupational status
		Personality: Test anxiety
		Subjective well-being: Sense of belonging to school
		Environmental awareness
		Environmental optimism
		Enjoyment of science
		Interest in broad science topics
		Instrumental motivation
		Science self-efficacy
		Epistemological beliefs
Schooling factors	School-related variables	School size
		Class size
		School ownership
		Shortage of educational material
		Creative extra-curricular activities
		Index of science specific resources
		Professional development of teachers
		Teachers’ participation
		Shortage of educational staff
		Proportion of teachers in school with bachelor’s degree
		Proportion of teachers in school with master’s degree
		Proportion of teachers in school with doctoral degree
		Proportion of all teachers fully certified
		Total number of all teachers at school
		Proportion of science teachers by all teachers
		Proportion of science teachers fully certified
		Proportion of science teachers with bachelor’s/master’s degree and a major in science
		Total number of science teachers at school
		Student-teacher ratio
		School policies for parental involvement
		Educational leadership
		Curricular development
		Instructional leadership
		Responsibility for curriculum
		Responsibility for resources
		School autonomy
		ICT resources
		Use of ICT at school in general
		Number of available computers per student at modal grade
		Proportion of available computers that are connected to the Internet
	Classroom-related variables	Disciplinary climate in science classes
		Teacher support in science classes of students’ choice
		Inquiry-based science teaching and learning practices
		Teacher-directed science instruction
		Comparison of science school lessons and additional instruction: Support
		Comparison of science school lessons and additional instruction: Structuredness of lessons
		Comparison of science school lessons and additional instruction: Structuredness of content
		Comparison of science school lessons and additional instruction: Teacher-student relation
		Teacher fairness
		Teacher behavior hindering learning
Parental factors	Parental beliefs and support	Parents’ perceived school quality
		Parents’ view on science
		Parents’ concerns regarding environmental topics
		Parents’ view on future environmental topics
		Parents’ emotional support
		Parents’ current support for learning at home
